# EGFR T790M Mutation Detection in Patients With Non-Small Cell Lung Cancer After First Line EGFR TKI Therapy: Summary of Results in a Three-Year Period and a Comparison of Commercially Available Detection Kits

**DOI:** 10.3389/pore.2022.1610607

**Published:** 2022-10-05

**Authors:** Eszter Bencze, Krisztina Bogos, Andrea Kohánka, László Báthory-Fülöp, Veronika Sárosi, Erzsébet Csernák, Nóra Bittner, Zsombor Melegh, Erika Tóth

**Affiliations:** ^1^ Department of Surgical and Molecular Pathology, National Tumour Biology Laboratory, National Institute of Oncology, Budapest, Hungary; ^2^ National Koranyi Institute of Pulmonology, Budapest, Hungary; ^3^ Faculty of Medicine, University of Pécs, Pécs, Hungary; ^4^ Department of Oncology Faculty of Medicine, Semmelweis University, Budapest, Hungary

**Keywords:** liquid biopsy, NSCLC, ctDNA, EGFR T790M mutation, AmoyDx super-ARMS EGFR mutation detection kit, cobas EGFR test v2

## Abstract

*EGFR* mutation in non-small cell lung cancer (NSCLC) offers a potential therapeutic target for tyrosine kinase inhibitor (TKI) therapy. The majority of these cases, however eventually develop therapy resistance, mainly by acquiring *EGFR* T790M mutation. Recently, third-generation TKIs have been introduced to overcome T790M mutation-related resistance. Cell free circulating tumor DNA (liquid biopsy) has emerged as a valuable alternative method for T790M mutation detection during patient follow up, when a tissue biopsy cannot be obtained for analysis. In this study, we summarized our experience with Super-ARMS EGFR Mutation Detection Kit (AmoyDx) on 401 samples of 242 NSCLC patients in a 3-year period in Hungary, comprising 364 plasma and 37 non-plasma samples. We also compared the performance of two commercially available detection kits, the cobas EGFR Mutation test v2 (Roche) and the Super-ARMS EGFR Mutation Detection Kit (AmoyDx). The same activating *EGFR* mutation was detected with the AmoyDx kit as in the primary tumor in 45.6% of the samples. T790M mutation was identified in 48.1% of the samples containing activating *EGFR* mutation. The detection rate of T790M mutation was not dependent on the DNA concentration of the plasma sample and there was no considerable improvement in mutation detection rate after a second, subsequent plasma sample. The concordance of *EGFR* activating mutation detection was 89% between the two methods, while this was 93% for T790M mutation detection. The AmoyDx kit, however showed an overall higher detection rate of T790M mutation compared to the cobas kit (*p* = 0.014). T790M mutation was detected at 29.8% of the patients if only plasma samples were available for analysis, while the detection rate was 70.2% in non-plasma samples. If the activating *EGFR* was detected in the plasma samples, the detection rate of T790M mutation was 42.4%. Although non-plasma samples provided a superior T790M mutation detection rate, we found that liquid biopsy can offer a valuable tool for T790M mutation detection, when a tissue biopsy is not available. Alternatively, a liquid biopsy can be used as a screening test, when re-biopsy should be considered in case of wild-type results.

## Introduction

Lung cancer is one of the most common malignant tumors with high mortality rate. Hungary was reported to have one of the highest incidence and mortality rates of lung cancer worldwide (1). The majority of the patients are smokers. In European studies, mutation in the *EGFR* gene exon 18, 19, 20 or 21 can be detected in 12%–17% of non-small cell lung cancers (NSCLC). These patients are predominantly non-smokers with a diagnosis of adenocarcinoma (2). EGFR tyrosine kinase inhibitor (TKI) therapy proved to be highly effective in these cases. The most common activating mutations are the point mutation L858R and a subset of in-frame deletions involving exon 19 (3). While the first-generation TKIs, such as gefitinib and erlotinib reversibly bind to EGFR, the binding and inhibition are irreversible at second-generation inhibitors, such as afatinib and dacomitinib. Despite this difference in their tyrosine-kinase binding ability, there is a similar pattern of acquired resistance, which usually occurs after 9–14 months (4). The resistance mechanism mainly involves gatekeeper *EGFR* mutations, activation of alternative signaling pathways through *MET* and *ERBB2* amplifications or activation of downstream MAPK or PI3K pathways (5). Among secondary gatekeeper *EGFR* mutations, T790M substitution mutation in exon 20 has emerged as the most common mechanism of acquired resistance (6). Most recently, third-generation EGFR TKIs, such as osimertinib have been introduced to overcome therapy resistance, which can evade *EGFR* T790M mutation by irreversibly binding to the mutated protein (7). This emphasizes the necessity of identifying patients with *EGFR* T790M mutation, as they may benefit from third-generation TKI therapy.

Tumor tissue biopsy has been conventionally used for tumor sampling and mutation analysis. However, it is not always possible to obtain a tissue sample when the tumor is difficult to access or a biopsy is hindered by the impaired health of the patient. Therefore, a less invasive method may be preferred to obtain tumor DNA in these cases. Cell-free circulating tumor DNA (ctDNA) regularly appears in the blood as a consequence of tumor cell apoptosis and necrosis, and has been reported to faithfully mirror the DNA content of the primary tumor of the same patient (8). Furthermore, it may better reflect the genetic heterogeneity of the tumor than a tissue biopsy (9). Hence, obtaining ctDNA from peripheral blood plasma emerged as a promising alternative to invasive tissue biopsies. Despite this fact, there are various factors which make detection of ctDNA difficult: the heavily fragmented nature and short half-life of ctDNA, low and widely variable plasma concentration levels, and the presence of background normal plasma DNA (10). This emphasizes the importance of employing a highly sensitive, reliable and accurate detection method. There are multiple diagnostic assays developed to assess *EGFR* status in blood plasma samples, including a number of PCR based systems, mass spectrometry, and next-generation sequencing (11). In this study, we summarized our results with the Amoydx Super-ARMS EGFR Mutation Detection Kit (Amoy Diagnostics Co., Ltd., Xiamen, China) test and we also compared the performance of the AmoyDx and the cobas EGFR Mutation test v2 (cobasv2; Roche Molecular Systems, Pleasanton, CA, United States) in peripheral plasma samples of NSCLC patients.

## Material and Methods

### Patients and Samples

Altogether 401 samples of 242 NSCLC patients, previously treated with first- or second-generation EGFR TKI therapy have been analyzed in the Molecular Pathology Laboratory of the National Institute of Oncology between 2019 and 2021. The number of cases in different sample types was as follows: 3 pleural effusions, 5 cell blocks, 6 cytology smears, 6 resection specimens, 17 lung biopsies and 364 blood samples. All the cytology and tissue samples contained viable tumor cells. The average number of blood samples per patient was 1.65; the maximum number was 6 blood samples per patient. Blood specimens and pleural effusions were collected and stored either in K2EDTA S-Monovette tubes (Sarstedt, Nümbrecht, Germany) at 4°C or in Cell-Free DNA BCT Streck tubes (Streck, La Vista, NE, United States) at room temperature, depending on the proximity of the sampling site. In the first case, liquid biopsies were processed within 2 h, while in the case of Streck tubes the time interval did not exceed 72 h.

The study was reviewed and approved by ETT-TUKEB, Health and Scientific Committee of Ministry of Human Resources of Hungary. Number of permission: IV/1792-4/2021/EKU.

### DNA Extraction

For an optimal yield and purity of ctDNA, plasma separation was performed in a two-step centrifugation protocol with a first centrifugation step of 3000×g for 10 min and a second step of 12,000×g for 10 min (12, 13). The ctDNA isolation started immediately after the plasma separation or after a maximum of 3-day storage at −80°C. Plasma samples between 4 and 6 ml were processed with the cobas ctDNA Sample Preparation Kit (Roche) following the manufacturer’s instructions. For preparation of pleural effusion samples, 9–17 ml of the samples were centrifuged at 1700×g for 10 min to remove cells and debris. The supernatant was used for the extraction of ctDNA with the same isolation protocol as for the plasma samples. For DNA extraction from small biopsies and cytological specimens with low tumor cell contents, we preferred the ReliaPrep DNA Clean-Up and Concentration System kit (Promega, Madison, WI, United States) to achieve a highly concentrated DNA yield. As this system contains only purifying reagents, the deparaffinization and lysis steps were performed with the corresponding reagents, according to the Maxwell RSC DNA FFPE protocol (Promega). The same kit was also used for formalin fixed paraffin embedded (FFPE) resection specimens. In each case, the tumor area was macro-dissected and one to three 5-µm sections of an FFPE specimen were processed. The concentration of the DNA samples was measured by the Qubit 4 fluorometer with the Qubit™ 1X dsDNA HS (High Sensitivity) Assay Kit (Thermo Fisher Scientific, Waltham, MA, United States) and stored at 4°C until further usage. For resection specimens, Promega, ReliaPrep DNA Clean-up and Concentration System kit was used.

### T790M Mutation Detection Methods

*EGFR* mutation detection was performed with the cobas *EGFR* Mutation test v2 on cobas z480 analyzer (Roche) and with the Super-ARMS EGFR Mutation Detection Kit (AmoyDx) on LightCycler 480 II instrument (Roche). All kits and devices were used as recommended by the manufacturer’s protocol. In the majority of cases, due to limited amount of DNA, only the AmoyDx kit was used as a standard method. When sufficient amount of ctDNA was available, we performed the test with both methods to compare the performances of the two cross-platform technologies.

### Statistical Analysis

McNemar’s test for paired proportions was used to calculate the differences of the concordance rates of the different methods. The correlation of DNA concentration and T790M mutation detection rate was calculated with the point-biserial correlation method. Significance was calculated with two-tailed t-tests or chi-square tests.

## Results

### T790M Mutation Detection Results With AmoyDx Test

Four plasma samples were not suitable for ctDNA analysis due to hemolysis. The result of 17 plasma samples and one resection specimen was invalid. The average DNA concentration according to the sample type is shown in Table 1. There were 9 cases where the ctDNA concentration was higher than 10 ng/μl.

**TABLE 1:** Average DNA concentration, number of samples and proportion of patients showing T790M mutation according to sample type.

Sample type (number of cases 397)	Average DNA concentration ng/μl (min-max)	Number and proportion of T790M positive samples	Proportion of patients with T790M mutation (%)
Pleural effusion (3)	11.6 (3.8–20)	2 (66.6%)	66.6
Cell blocks (5)	2.8 (1.3–4.6)	3 (60%)	60
Cytology smears (6)	11.9 (0.1–44.8)	2 (33.3%)	33.3
Biopsy (17)	16.6 (1.1–60)	15 (88%)	88
Resection (6)	56.9 (5.1–155)	4 (66.6%)	66.6
Plasma (360)	3.9 (0.1–65)	61 (16.9%)	29.5

We also assessed if the activating *EGFR* mutation of the corresponding primary tumors, previously analyzed with cobas *EGFR* Mutation test v2, could be detected in the sample. With the AmoyDx kit, we detected the same activating *EGFR* mutation which was seen in the primary tumors in 181 of 397 samples (45.6%). Eighty-seven of these 181 samples (48.1%) contained T790M mutation. Out of these 87 cases, T790M mutation was detected with the AmoyDx kit in the first blood samples of the patients in 50 cases and in a subsequent second blood sample in a further 32 cases. In 4 cases, only the third blood sample was T790M positive and we had only 1 case when only the fourth blood sample showed a positive result. Further repetition of the sampling did not improve the detection rate, as we did not detect any T790M mutation after the fourth sample, when the previous samples were negative.

Considering the sample type, T790M mutation was detected at 70.3% of the patients if non-plasma samples were used, while it was detected at only 29.8% of the patients if only plasma samples were analyzed. Although the number of non-plasma samples was relatively low, the difference was significant (*p* < 0.0001) (Table 2). Theoretically, the lower T790M detection rate in the plasma samples could be explained by the absence of circulating tumor DNA in the sample. Nevertheless, when we included only those samples in the analysis, where the primary *EGFR* mutation was also identified, hence confirming the presence of ctDNA in the sample, the difference remained significant (*p* = 0.002444) (Table 3).

**TABLE 2:** Results of T790 mutation analysis of the samples of all the patients, according to the sample type (chi-square *p* < 0.0001).

Sample type	T790 mutation analysis results (number of patients)	
	T790 wild type	T790M mutant
Non-plasma	11 (29.7%)	26 (70.3%)
Plasma	144 (70.2%)	61 (29.8%)

**TABLE 3:** Results of T790 mutation analysis when the primary activating *EGFR* mutation was also detected in the sample, according to the sample type (chi-square *p* = 0.00244).

Sample type	T790 mutation analysis results (number of cases)	
	T790 wild type	T790M mutant
Non-plasma	11 (29.7%)	26 (70.3%)
Plasma	83 (57.6%)	61 (42.4%)

**FIGURE 1:**
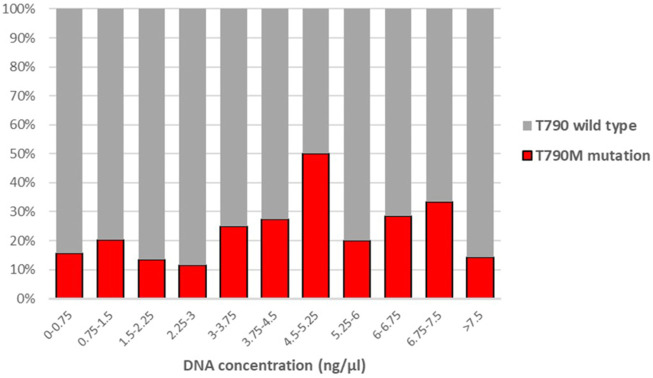
Correlation between the proportion of T790M positive cases and DNA concentration. (Point-biserial correlation *p* = 0.339).

We also examined whether the T790M mutation detection rate of the AmoyDx kit correlated with the DNA concentration. Figure 1 shows the T790M mutation frequency in relation to the DNA concentration. There was no significant correlation between the DNA concentration and the detection rate (*p* = 0.339), and the proportion of positive cases did not depend on the DNA concentration.

The type of the primary *EGFR* mutations in relation to the presence of T790M mutation is shown in Table 4. T790M mutation was most frequent in exon 19 del and exon 21 mutant cases. Frequency of T790M mutation was roughly similar in *EGFR* exon 19 del and exon 21 mutant groups, 49 of 94 (52.1%) and 36 of 75 (48%), respectively.

**TABLE 4:** Occurrence of T790M mutation in different *EGFR* mutant groups.

Type of primary *EGFR* mutation	Number of T790M positive samples/total
Exon 19 del	49/94
Exon 20 ins	1/1
Exon 21 L858R	33/68
Exon 21 L861Q	2/4
Exon 21 L861Q and exon 20 S768I	1/3
Exon 18 G719X	0/7
Exon 18 G719X and exon 20 S768I	0/2
Exon 18 G719X and exon 21 L861Q	1/2

### Comparison of the results of cobas *EGFR* mutation test v2 and AmoyDx Super-ARMS test

Eighty-six plasma samples had sufficient amount of DNA for analysis with both methods. The same amount of DNA was used for comparison. These cases were also used to assess the concordance rate between the plasma samples and the previously obtained tissue samples from the same patient.

In 73 plasma samples, 85% of all cases, we obtained the same result with both tests. With the AmoyDx Super-ARMS test, 43 cases showed the same activating *EGFR* mutation, which was detected previously in the primary tumor (50.5%) and 20 out of these 43 *EGFR* mutant cases also showed T790M positivity. With cobas test, 40 cases (46%) showed the same activating *EGFR* mutation, which was previously detected in the primary tumor and T790M mutation was present in 14 out of these cases. Overall, 20/86 samples (23%) tested with the AmoyDx kit were T790M mutation positive vs. 14/86 (16%) with the cobas kit. The higher T790M mutation detection rate of the AmoyDx kit compared to the cobas kit was statistically significant (*p* = 0.014). In six samples, the T790M mutation was only detectable with the AmoyDx kit. The concordance rate of *EGFR* activating mutation detection between the two methods was 89%, while this was 93% for T790M mutation detection.

Seventy-one plasma samples analyzed by both AmoyDx and cobas tests had a previous tissue sample with an available *EGFR* mutation analysis result. Among these cases, four samples had an *EGFR* activating mutation detected only by the AmoyDx test, while cobas test showed a wild-type result in these cases. In one case, the same double mutation (exon 18 G719x, exon 20 S768I) was detected in the primary tumor and with AmoyDx test in the plasma sample, but the cobas test detected only the exon 20 S768I mutation. In one case, where the primary lung tumor showed exon 21 L858R mutation, the same mutation was detected with cobas test in the ctDNA, but the AmoyDx test showed an exon 20 ins. We interpreted the AmoyDx result as being false positive, since the primary tissue sample analysis showed an exon 21 L858R mutation only, although this could also be explained by tumor heterogeneity. In addition, in one case, we detected the same *EGFR* exon 19 del mutation with cobas test as in the primary tumor sample, but the AmoyDx gave a wild-type result. The concordance rate between the primary tissue samples and the ctDNA testing platforms is shown in Table 5. Although the AmoyDx test showed a slightly better performance in detecting the primary activating *EGFR* mutation in case of exon 19 del, exon 18 mut and exon 21 mut, the difference between the two platforms was not statistically significant (*p* = 0.563, *p* = 0.317, *p* = 0.157, respectively). The overall concordance rate with the tissue sample result was 86% for the cobas and 88% for the AmoyDx kit, the difference of which was again not statistically significant (*p* = 0.667).

**TABLE 5:** Concordance between the primary tissue samples and the plasma ctDNA results for *EGFR* activating mutations.

	Concordance		Probability of difference in concordance rates
	Cobas	AmoyDx	
exon 19 del	50/71 (70%)	52/71 (73%)	*p* = 0.563
exon 18 mut	67/71 (94%)	68/71 (96%)	*p* = 0.317
exon 21 mut	63/71 (89%)	65/71 (92%)	*p* = 0.157
exon 20 mut	64/71 (90%)	64/71 (90%)	n.a

## Discussion

While T790M mutation occurs in less than 5% of untreated *EGFR* mutant lung adenocarcinomas, about 50%–70% of the *EGFR* mutated tumors develop T790M mutation as an acquired resistance if treated with first-generation TKIs (14). As a minimally invasive test, assessment of ctDNA in plasma samples is a valid and cost-effective alternative of tissue biopsies and identifies a large proportion of *EGFR* T790M mutations responsible for therapy resistance. In this study, we presented the results of *EGFR* T790M mutation testing in our laboratory over a 3-year period. Our results are in accordance with the literature data: as we detected T790M mutation in 48.1% of the samples, where the primary *EGFR* mutation could also be detected, confirming the presence of ctDNA in the sample (15). The AmoyDx test routinely used by us showed a higher detection rate of T790M mutation compared to the cobas test, and the difference was significant (*p* = 0.014). The concordance rate between the two tests was 93% in detecting T790M mutation and 89% in detecting the activating *EGFR* mutation. Compared to the previous tissue samples of the patients, the AmoyDx kit had a higher concordance rate than the cobas test kit in detecting activating *EGFR* mutations (86% vs. 88%), but this difference was not significant (*p* = 0.667). Overall, the detection rate of the AmoyDx test was slightly better than of the cobas test in our hands. However, cobas test is a very simple automated detection method with one of the widest *EGFR* mutation coverage among PCR based methods (42 mutations of exon 18, 19, 20, 21). Nevertheless, it was already shown previously that its sensitivity is inferior compared to ARMS techniques (allele specific polymerase chain reaction), like the AmoyDx test which also covers 42 mutations of exon 18, 19, 20 and 21, or digital PCR methods (16).

Our data suggest that plasma testing is useful in patients with *EGFR* T790M resistance mutations where continuous monitoring is recommended, especially if a tissue biopsy is not available. According to recent studies, in about 40% of relapsed cases it is not possible to obtain tumor tissue samples suitable for molecular analysis (17). In our hands with the AmoyDx test, T790M mutation frequency was 42.4% in those plasma samples where the activating *EGFR* mutation was detected. At the same time, we found that the detection of T790M mutation was not dependent on ctDNA concentration of the plasma samples. Furthermore, when multiple blood samples of a patient were sequentially taken at different points in time, the detection rate did not improve considerably after two repeated blood samples.

Although the number of non-plasma samples was very small in our study, comprising only 9.2% of the samples, the detection rate of T790M mutation was much higher than in non-plasma samples. This is in keeping with others studies on T790M testing. Tissue biopsy is still considered the gold standard for *EGFR* mutation analysis and up to 30% of the negative plasma samples for T790M mutation can have a positive result in a subsequent tissue biopsy (18). According to the results of Pereira et al, re-biopsy increased the detection rate of T790M mutation with 17% (19). The most probable cause of the higher detection rate of T790M in tissue samples is a higher tumor DNA content and a higher mutant allele frequency. Consequently, ctDNA mutational analysis could potentially be used as a triage test, where patients with a negative sample could undergo a further tissue biopsy. Recently, it has been demonstrated that using ctDNA as a triage test results in a superior T790M detection rate than tissue biopsy alone (20).

Re-biopsy rate can be considerably high in some practices. According to a recent Japanese publication on 120 patients with acquired resistance to EGFR-TKIs, re-biopsy was performed on 109 patients, with an implementation rate of 90.8%. However, re-biopsy is still very rare in our practice compared to the literature data. Although liquid biopsy alone is still less costly than tissue biopsy, considering its costs and effects directly relating to testing (20), re-biopsy should be considered more frequently in our practice in case of disease progression after first- and second-line *EGFR* TKI therapy.

## Data Availability

The raw data supporting the conclusion of this article will be made available by the authors, without undue reservation.
